# HSCARG Negatively Regulates the Cellular Antiviral RIG-I Like Receptor Signaling Pathway by Inhibiting TRAF3 Ubiquitination *via* Recruiting OTUB1

**DOI:** 10.1371/journal.ppat.1004041

**Published:** 2014-04-24

**Authors:** Yanyan Peng, Ruidan Xu, Xiaofeng Zheng

**Affiliations:** 1 State Key Lab of Protein and Plant Gene Research, School of Life Sciences, Peking University, Beijing, China; 2 Department of Biochemistry and Molecular Biology, School of Life Sciences, Peking University, Beijing, China; Kantonal Hospital St. Gallen, Switzerland

## Abstract

RIG-I like receptors (RLRs) recognize cytosolic viral RNA and initiate innate immunity; they increase the production of type I interferon (IFN) and the transcription of a series of antiviral genes to protect the host organism. Accurate regulation of the RLR pathway is important for avoiding tissue injury induced by excessive immune response. HSCARG is a newly reported negative regulator of NF-κB. Here we demonstrated that HSCARG participates in innate immunity. HSCARG inhibited the cellular antiviral response in an NF-κB independent manner, whereas deficiency of HSCARG had an opposite effect. After viral infection, HSCARG interacted with tumor necrosis receptor-associated factor 3 (TRAF3) and inhibited its ubiquitination by promoting the recruitment of OTUB1 to TRAF3. Knockout of HSCARG attenuated the de-ubiquitination of TRAF3 by OTUB1, and knockdown of OTUB1 abolished the effect of HSCARG. HSCARG also interacted with Ikappa-B kinase epsilon (IKKε) after viral infection and impaired the association between TRAF3 and IKKε, which further decreased the phosphorylation of IKKε and interferon response factor 3 (IRF3), thus suppressed the dimerization and nuclear translocation of IRF3. Moreover, knockdown of TRAF3 dampened the inhibitory effect of *IFN-β* transcription by HSCARG, suggesting that TRAF3 is necessary for HSCARG to down-regulate RLR pathway. This study demonstrated that HSCARG is a negative regulator that enables balanced antiviral innate immunity.

## Author Summary

Innate immunity is critical for the host to defeat pathogen invasion, and the production of interferon (IFN) is the core of the cellular antiviral response, this is mediated by the Toll-like receptor (TLR) and RIG-I like receptor (RLR) signaling pathways in most cell types. As aberrant activity of the immune response leads to immune-deficiency or autoimmune disease, identification of the regulators involved in immune balance is particularly important. Accumulating evidence shows that ubiquitination plays a key role in regulating virus-triggered IFN production to assure that the antiviral response is modulated properly. Here, we demonstrated that HSCARG is a novel negative regulator in the precise control of antiviral innate immunity. HSCARG inhibited IFN-β production by suppressing TRAF3 ubiquitination with the help of OTUB1, leading to disassociation of downstream IKKε and impairment of IRF3 activity. As the pivot of the TLR, RLR, and non-canonical NF-κB pathways, TRAF3 is an extremely versatile immune regulator. Our study sheds light on the mechanism of specificity and diversity achievement in the complicated regulation of TRAF3 activity, suggesting that HSCARG is a potential target for the treatment of inflammatory and autoimmune diseases.

## Introduction

Innate immunity is the first line of defense for organisms to defeat disease-causing pathogens, such as viruses, bacteria, and parasites. The production of interferons (IFNs) is the core of the cellular antiviral response [1]. IFNs are secreted by virus-infected cells and are recognized by the interferon-α/β receptor in the cell membrane; they then promote the transcription of a series of antiviral genes through the Janus kinase-signal transducer and activator of transcription (JAK-STAT) pathway [2]. The induction of Type I IFNs is governed by both of the transcription factors NF-κB and IRF3/IRF7 that are activated by pattern recognition receptors (PRRs) [3], [4].

Toll-like receptors (TLRs) and RIG-I like receptors (RLRs) are the main PRRs involved in the cell-specific regulation of Type I IFNs [5]. RLRs include retinoic acid inducible gene I (RIG-I) [6] and melanoma differentiation-associated gene 5 (MDA5) [7], which recognize viral RNA through the RNA helicase domain (RLD) and then recruit mitochondrial-associated virus stimulator (MAVS, also known as IPS-1, Cardif, or VISA) [8]–[11] through CARD-CARD interaction. MAVS further recruits tumor necrosis receptor-associated factor 3 (TRAF3) [12] and results in TRAF3 K63-linked auto-ubiquitination, which provides docking sites for the TANK binding kinase 1/I kappa-B kinase epsilon (TBK1/IKKε) complex [13], [14]. This complex undergoes auto-phosphorylation-mediated activation, subsequently phosphorylates its substrate IRF3/7, and then induces IRF3/IRF7 to form homodimers or heterodimers that translocate into the nucleus to bind the interferon promoter positive regulatory domains [15], [16]. Besides, after TLR ligand stimulation, NF-κB is activated as well and starts type I IFN transcription through the MyD88-dependent or TRIF-dependent pathway [17]. More importantly, an aberrant RLR pathway is associated with multiple inflammatory diseases, so the intensity and duration of the antiviral response needs to be precisely regulated to prevent inflammation or autoimmune disease.

Increasing evidence has shown that ubiquitination and de-ubiquitination of adaptor proteins such as RIG-I, MAVS, TRAF3, and IRF3 are widely involved in the precise regulation of antiviral response activity [18]–[22].TRAF3 belongs to the Ring finger ubiquitin E3 ligase family TRAF and is a versatile immune regulator with multiple functions in several signaling pathways [23]. It contributes to type I IFN production while attenuating mitogen-activated protein kinase activation and the alternative NF-κB signaling pathway through the recruitment of different complexes and various modes of auto-ubiquitination [14]. Triad3A is reported to conjugate TRAF3 with a K48-linked polyubiquitin chain, while it seems to generate a K63-linked polyubiquitin chain for itself [21], [24]. So far, several deubiquitinases such as DUBA, OTUB1/2, UCHL1, and OTUD7B have been reported to cleave TRAF3 ubiquitin chains in response to viral infection [25]–[28]. However, how these regulators cooperate to orchestrate TRAF3 activity in specific contexts remains elusive. Besides, some novel proteins involved in the complicated fine-tuning of TRAF3 activity await discovery.

HSCARG (also named NmrA-like family domain-containing protein 1 or NMRAL1) is a newly-identified NADPH sensor and negative regulator of NF-κB [29]. It contains a Rossmann-fold in the N terminus and forms an asymmetrical dimer with only one subunit occupied by one NADP molecule [30]. In response to decreases in the NADPH/NADP^+^ ratio within cells, HSCARG interacts with argininosuccinate synthetase (AS) more potently, resulting in stronger inhibition of AS activity and NO production [31]. Besides, HSCARG negatively regulates TNFα-stimulated NF-κB activity by suppressing IKKβ phosphorylation and further blocking the degradation of IκBα [29]. Since NF-κB is an essential transcription factor in the cellular antiviral response, and RNA-seq analysis of *HSCARG* wild-type and *HSCARG^−/−^* HCT116 cells showed that HSCARG affects the mRNA level of several adaptor proteins in the RLR pathway, such as *TBK1*, *RIG-I*, *MDA5*, and *MITA*, we set out to investigate the function of HSCARG in cellular antiviral pathway. Here, we found that HSCARG dampens Sendai virus induced RLR signaling pathway activity through repressing K63-linked ubiquitination of TRAF3. This decreases IFN-β production and attenuates cellular antiviral response to prevent excessive inflammation.

## Results

### HSCARG negatively regulates the cellular antiviral response

**Figure 1:**
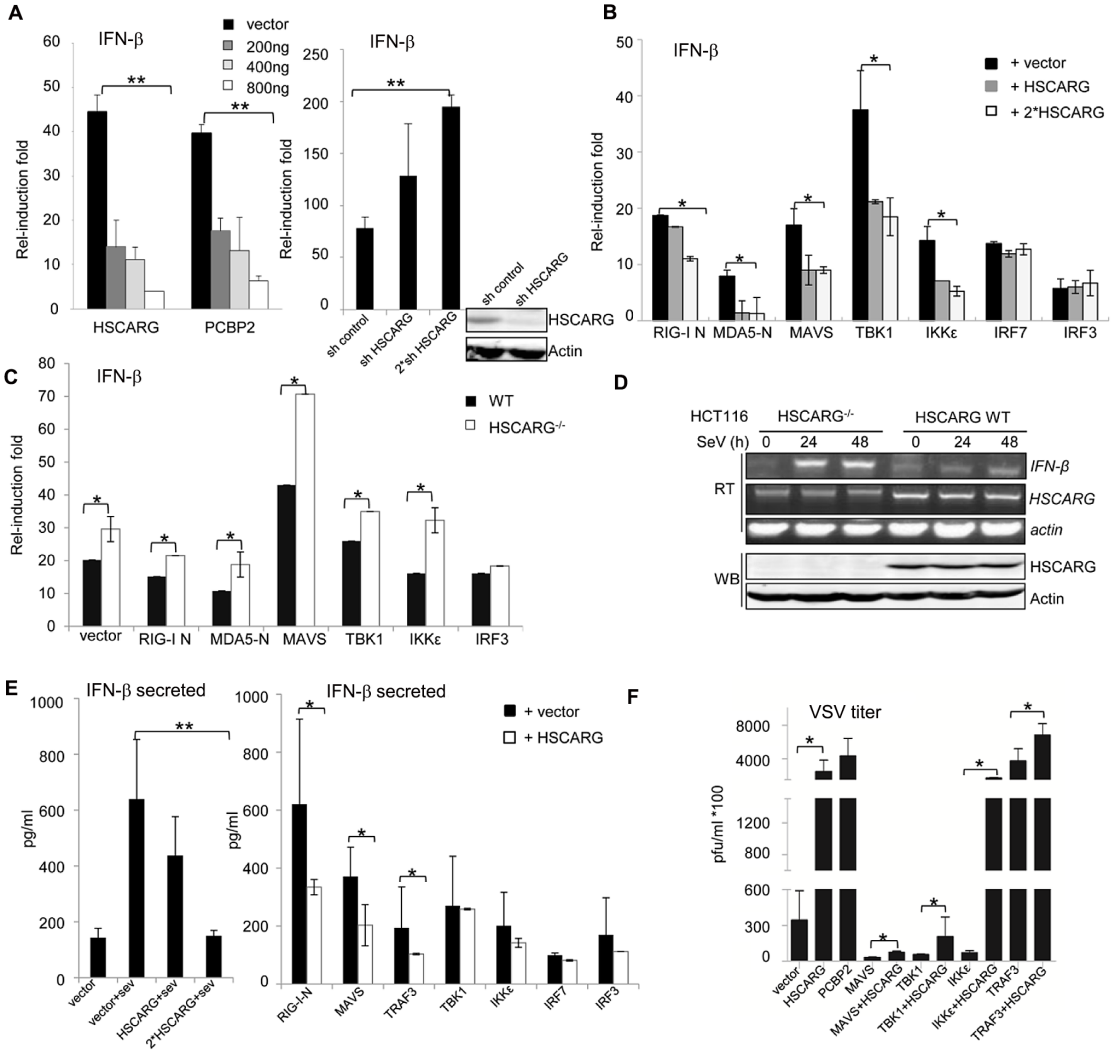
HSCARG negatively regulates cellular antiviral response. (A) HSCARG down-regulates *IFN-β* reporter activity. HEK293T cells (1×10^5^) were transfected with 200 ng *IFN-β* reporter plasmid, 20 ng pRL-TK1 control plasmid, and increasing dose of HSCARG (200, 400 and 800 ng), the positive control PCBP2 (200, 400 and 800 ng), or HSCARG shRNA plasmid (300 and 600 ng), and infected with SeV (40 HAU/ml) for 18 h, and then luciferase reporter assay was performed. The induction folds are presented relative to the luciferase activity in the control cells transfected with the empty vector. (B) HSCARG suppresses the *IFN-β* activity mediated by the RLR adaptors. HEK293T cells transfected with plasmids encoding RIG-I (N terminal), MDA5 (N terminal), MAVS, TBK1, IKKε, IRF7, IRF3, with vector or increasing dose of HSCARG, were infected with SeV (40 HAU/ml) for 18 h, and then the *IFN-β* reporter activity was examined. (C) The induction of *IFN-β* is increased in *HSCARG* ^−/−^ HEK293T. The wild-type and *HSCARG* ^−/−^ cells were transfected with the same amount of plasmids encoding RIG-I N (N terminal), MDA5-N (N terminal), MAVS, TBK1, IKKε, IRF3, and the *IFN-β* reporter activity was assessed as described above. (D) SeV infection induces more mRNA of *IFN-β* in *HSCARG* ^−/−^ HCT116 cells. Cells (1×10^5^) were harvested at 0, 24, and 48 h after SeV infection and RT-PCR was performed to monitor the *IFN-β* mRNA levels. The band of *HSCARG* mRNA in the *HSCARG* ^−/−^ cells is larger and thus its mobility is slower in the gel compared to the native *HSCARG* mRNA. (E) HSCARG suppresses IFN-β secretion in media supernatant. HEK293T cells (1×10^5^) transfected with increasing dose of HSCARG (left panel), or indicated plasmids together with vector or HSCARG (right panel) were treated with SeV (40 HAU/ml) for 18 h, and then ELISA was performed to detect the secreted IFN-β level in supernatant. (F) HSCARG promotes VSV proliferation. HEK293T cells (1×10^5^) were transfected with indicated plasmids as shown in the horizontal axis with or without HSCARG. 24 h later, cells were infected with VSV at MOI = 1 for 1 h, and then plaque assay was performed to measure virus titer. Experiments were performed at least three times with similar results. The bar graphs show mean±S.D. of triplicates from one representative experiment. *p<0.05, **p<0.01.

To investigate whether HSCARG functions in the cellular antiviral response, we assessed its effect on the activity of *IFN-β* promoter with a luciferase reporter assay. We found that HSCARG dose-dependently decreased the *IFN-β* reporter activity induced by Sendai virus (SeV) infection (Figure 1A), similar to the positive control PCBP2 which promotes MAVS degradation by recruiting the HECT ubiquitin ligase AIP4 thus repressing *IFN-β* activity [20]. Consistently, knockdown of HSCARG strongly enhanced the *IFN-β* promoter activity (Figure 1A, right). STK38 [20] was used as a negative control to confirm that the inhibitory effect of HSCARG is specific in the luciferase reporter assay system (Fig. S1A). Next, in order to test the effect of HSCARG on various components of the RLR pathway, we respectively transfected HEK293T cells with plasmids expressing RIG-I-N (N indicates the amino-terminal CARD domain; the full version is in a self-repressed state at rest), MDA5-N (same as RIG-I-N), MAVS, TBK1, IKKε, IRF3, IRF7 with or without HSCARG, and measured the *IFN-β* promoter activity. The reporter assay results showed that HSCARG inhibited the IFN-β activity induced by almost all the components upstream of IRF3/7 (Figure 1B), without affecting their stability (Figure S1B); whereas the absence of HSCARG significantly increased the RLR adaptors-mediated *IFN-β* promoter activity (Figure 1C). Furthermore, we examined the mRNA level of *IFN-β* in wild-type and *HSCARG* ^−/−^ HCT116 cells. As expected, cells without HSCARG exhibited an increased *IFN-β* mRNA level in response to viral infection (Figure 1D). Besides, we evaluated the secreted IFN-β level in the supernatant medium by ELISA and found that HSCARG clearly suppressed the IFN-β production and secretion triggered by SeV infection in a dose-dependent manner (Figure 1E, left), as well as essential RLR adaptors-mediated IFN-β production (Figure 1E, right). Finally, we performed the plaque assay in an attempt to detect the physiological importance of HSCARG in the cellular antiviral response. The results showed that cells overexpressing HSCARG impaired the antiviral response mediated by MAVS, TBK1, TRAF3 and IKKε, decreased the resistance to vesicular stomatitis virus (VSV) infection, and led to increased VSV propagation, which was consistent with the PCBP2 positive control (Figure 1F). Taking all the data together, HSCARG significantly inhibits the cellular antiviral response mediated by the RLR signaling pathway.

### HSCARG interacts with TRAF3 and suppresses TRAF3 K63-linked ubiquitination

As reported previously, HSCARG is a negative regulator of NF-κB that is important for *IFN-β* transcription. Therefore, it was necessary to detect whether HSCARG is solely dependent on NF-κB to accomplish its negative regulatory role in IFN-β production. We blocked NF-κB activation by knocking down p65 with a cocktail of p65 siRNA or using the specific NF-κB inhibitor, PDTC, and then assessed whether HSCARG lost its inhibitory effect on *IFN-β* promoter activity. The reporter assay results showed that blocking NF-κB activity by knocking down p65 only slightly impaired the inhibitory effect of HSCARG on the *IFN-β* and *ISRE* reporters (Figure S2A). Besides, blocking NF-κB activity with PDTC gave consistent results without affecting *ISRE* activity (Figure S2B). These data suggested that rather than *via* the NF-κB pathway, HSCARG down-regulates IFN-β by regulating IRF3 activation.

**Figure 2:**
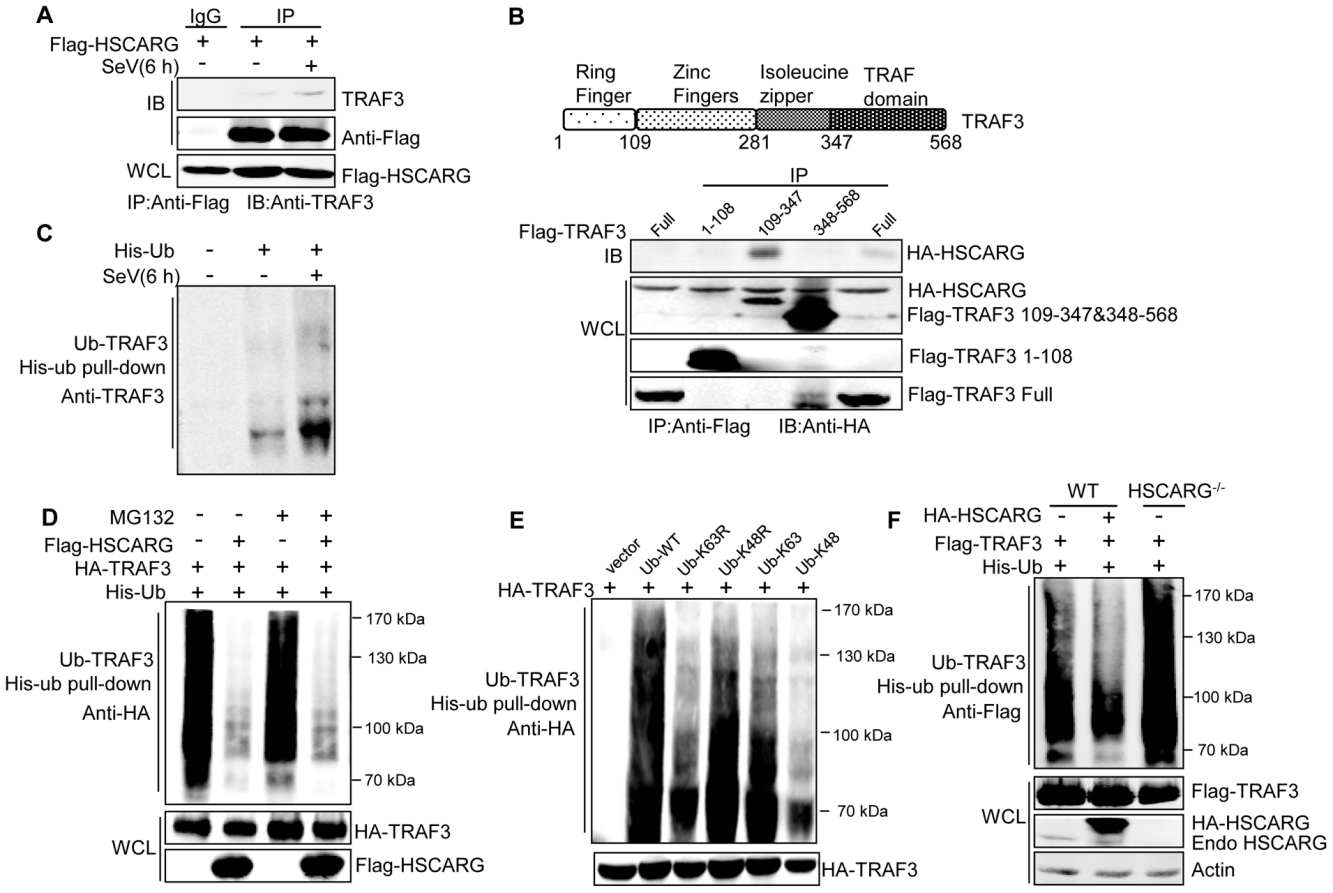
HSCARG interacts with TRAF3 and inhibits TRAF3 K63-linked ubiquitination. (A) HSCARG interacts with endogenous TRAF3. HEK293T cells were transfected with Flag-HSCARG for 24 h, and then infected with SeV (40 HAU/ml) for 6 h. Cell lysates were IP with anti-Flag or control IgG followed by IB with antibodies against TRAF3 and Flag. (B) Mapping of the TRAF3 domains for HSCARG binding. HEK293T cells were transfected with HA-HSCARG and Flag-TRAF3 1–108, 109–347, 348–568 truncation constructs, Co-IP was performed with anti-Flag antibody followed by IB with anti-HA antibody. The protein expression level was shown in the bottom. A diagram for TRAF3 truncations was shown on top. (C) Viral infection promotes endogenous TRAF3 ubiquitination. HEK293T cells were transfected with His-ubiquitin and infected with SeV for 6 h, and then His-ubiquitin pull-down analysis was performed followed by IB with antibody against TRAF3 to examine the level of endogenous TRAF3 ubiquitination. (D, E) TRAF3 is mainly modified with K63-linked ubiquitination after viral infection. HEK293T cells were transfected with HA-TRAF3, His-ubiquitin, together with or without Flag-HSCARG (D), or with various ubiquitin mutants *K63R*, *K48R*, *K63*, and *K48* (E). 24 h later, cells were treated with MG132 (20 ng/ml) and SeV (40 HAU/ml) for 6 h (D), and then subjected to His-ubiquitin pull-down and IB with anti-HA to monitor the ubiquitination of TRAF3. Here, *K63R* or *K48R* means that only Lysine63 or Lysine48 residue was mutated to arginine, *K63* or *K48* means only Lysine63 or Lysine48 residue remains unchanged while the other lysines were all mutated to arginines. (F) HSCARG inhibits TRAF3 ubiquitination. Wild-type and *HSCARG* ^−/−^ HEK293T cells transfected with Flag-TRAF3, His-ubiquitin, or in combination with HA-HSCARG were subjected to His-ubiquitin pull-down and IB with anti-Flag to examine the effect of HSCARG on TRAF3 ubiquitination.

Because IRF3 nuclear translocation is mainly governed by activating the RLR signaling pathway, in an attempt to test our hypothesis, co-immunoprecipitation (Co-IP) analysis was performed to identify the target proteins of HSCARG in the RLR pathway (Figure S3A). The results revealed that HSCARG interacted most potently with TRAF3 (Figure S3A), and this association was confirmed under physiological condition in which the interaction was enhanced by SeV infection (Figure 2A). Furthermore, the essential domain of TRAF3 that binds with HSCARG was delineated by using a series of TRAF3 truncation constructs, and the zinc finger and isoleucine zipper domains of TRAF3 were found to be the crucial regions responsible for the association of TRAF3 with HSCARG (Figure 2B).

It is well-known that TRAF3 is modified with a poly-ubiquitin chain to provide a scaffold for complex formation; this promoted us to study the effect of HSCARG on TRAF3 ubiquitination. First, we found that SeV infection increased the level of endogenous TRAF3 ubiquitination (Figure 2C). To explore the type of TRAF3 ubiquitin chains in response to early-phase viral infection, HEK293T cells transfected with TRAF3 were treated with or without the proteasome inhibitor MG132, and the ubiquitination level of TRAF3 was monitored. Compared to the control, MG132 treatment did not change the TRAF3 ubiquitination level (Figure 2D), suggesting that TRAF3 barely undergoes K48-linked ubiquitination at 6 h after viral infection. In addition, we constructed various ubiquitin mutants including *K63R*, *K48R*, *K63*, and *K48* to determine the TRAF3 poly-ubiquitin chain type. The level of TRAF3 ubiquitination evidently decreased when lysine 63 was mutated to arginine (*K63R*), but showed no significant change when lysine 48 was mutated to arginine (*K48R*) (Figure 2E). These data demonstrated that TRAF3 is mainly modified with K63-linked ubiquitination to transduce signals from MAVS to TBK1/IKKε in the early phase of viral infection. We further investigated whether HSCARG affected TRAF3 ubiquitination. His-ubiquitin pull-down assay showed that HSCARG markedly decreased TRAF3 ubiquitination. Consistently, TRAF3 poly-ubiquitination increased intensely in *HSCARG* ^−/−^ cells (Figure 2F). In summary, these results demonstrated that HSCARG associates with TRAF3 and suppresses the K63-linked ubiquitination of TRAF3.

### HSCARG cooperates with OTUB1 to inhibit TRAF3 ubiquitination

**Figure 3:**
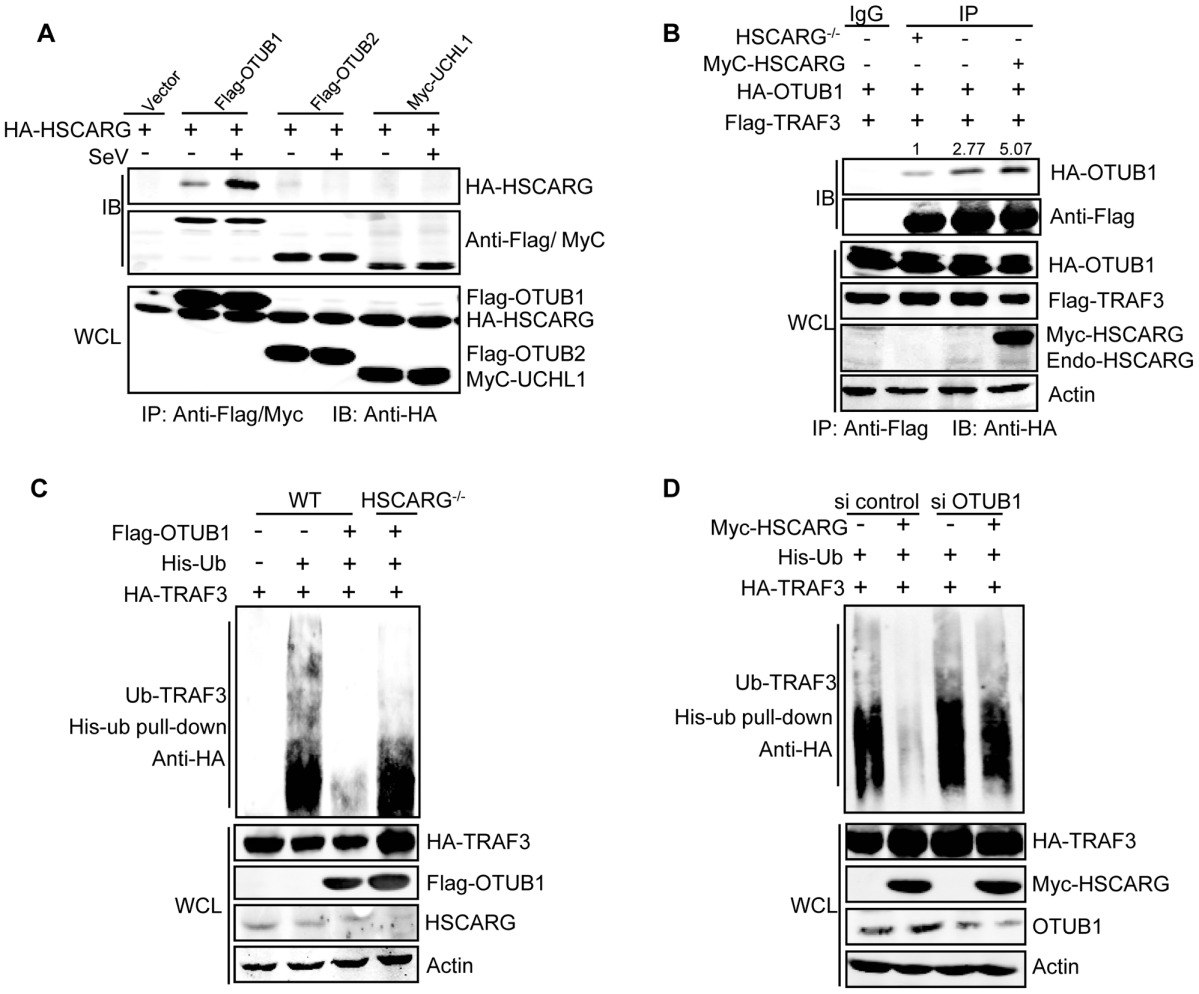
HSCARG and OTUB1 cooperatively inhibit TRAF3 ubiquitination. (A) HSCARG interacts with OTUB1. HEK293T cells transfected with indicated plasmids were infected with or without SeV (40 HAU/ml) for 6 h. Co-IP analysis was performed with anti-Flag or anti-Myc antibody followed by IB with anti-HA antibody. (B) HSCARG promotes TRAF3-OTUB1 interaction. *HSCARG* ^−/−^ and wild-type HEK293T cells were transfected with Flag-TRAF3, HA-OTUB1, or together with Myc-HSCARG. Co-IP was then performed with anti-Flag and IB with anti-HA to examine the effect of HSCARG on the TRAF3-OTUB1 complex. Quantification of HA-OTUB1 was performed using Odyssey Infrared Imaging System and software Odyssey V3.0. The data was normalized to the enriched protein. (C) OTUB1 predominantly loses its de-ubiquitination ability in *HSCARG* ^−/−^ cells. The wild-type and *HSCARG* ^−/−^ HEK293T cells were transfected with HA-TRAF3, His-ubiquitin, together with or without Flag-OTUB1, and then subjected to His-ubiquitin pull-down analysis and IB with anti-HA to measure TRAF3 ubiquitination level. (D) HSCARG relies on OTUB1 to inhibit TRAF3 ubiquitination. HEK293T cells were transfected with OTUB1 siRNA (40 nM), negative control, and HA-TRAF3, His-ubiquitin with or without Myc-HSCARG as indicated. 72 h later, His-ubiquitin pull-down was performed followed by IB with anti-HA to detect TRAF3 ubiquitination level.

Because HSCARG does not belong to any known deubiquitinase family, it is reasonable to hypothesize that HSCARG works through recruiting a deubiquitinase to cleave the TRAF3 ubiquitin chain. We first screened the reported deubiquitinases for TRAF3 and found that HSCARG interacted with OTUB1 more potently than OTUB2, UCHL1, OTUD7B, and USP25 (Figure 3A, Figure S3B), and this interaction was enhanced by viral infection (Figure 3A). Hence, we focused on OTUB1 to further elucidate the regulatory mechanism of HSCARG in TRAF3 ubiquitination. Co-IP showed that overexpression of HSCARG enhanced the interaction of TRAF3 with OTUB1, whereas in *HSCARG* ^−/−^ cells, this association was severely attenuated (Figure 3B). These findings indicated that HSCARG promotes the recruitment of OTUB1 to TRAF3. Furthermore, OTUB1 lost a majority of its de-ubiquitination function in *HSCARG* ^−/−^ cells (Figure 3C), and its inhibition of *IFN-β* activity was also attenuated when HSCARG was knocked out (Figure S3C), while in cells with depleted OTUB1, HSCARG no longer inhibited TRAF3 ubiquitination (Figure 3D). These results indicated that OTUB1 and HSCARG function cooperatively in down-regulating TRAF3 ubiquitination.

### HSCARG interacts with IKKε and impairs the formation of the TRAF3 and IKKε complex

**Figure 4:**
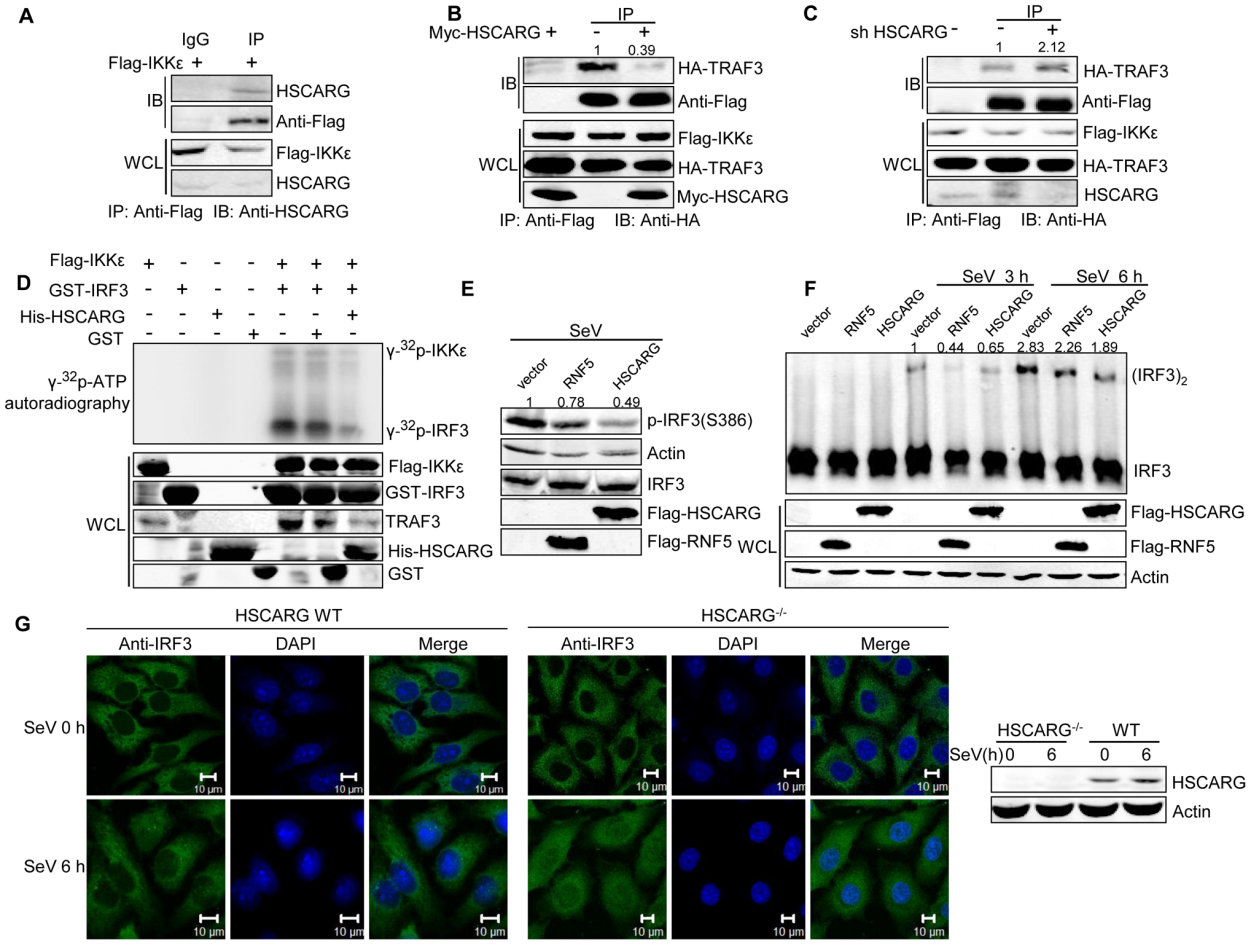
HSCARG interacts with IKKε and blocks the formation of TRAF3-IKKε complex. (A) Endogenous HSCARG interacts with IKKε. HEK293T cells transfected with Flag-IKKε were IP with anti-Flag followed by IB with anti-HSCARG or anti-Flag. (B, C) HSCARG blocks the interaction between IKKε and TRAF3. HEK293T cells transfected with Flag-IKKε, HA-TRAF3 with or without Myc-HSCARG (B), or HSCARG shRNA (C) were subjected to Co-IP analysis to examine the effect of HSCARG on the IKKε-TRAF3 interaction. (D) HSCARG decreases IKKε and IRF3 phosphorylation *in vitro*. Flag-IKKε was enriched from cell lysate with anti-Flag, GST, GST-IRF3 (131–426) and His-HSCARG were purified from *E.coli*, and *in vitro* phosphorylation was performed as described in the Methods. Anti-TRAF3 antibody was used to detect the existence of TRAF3 in the enriched Flag-IKKε. (E) HSCARG impairs IRF3 phosphorylation. HEK293T cells transfected with vector or Flag-RNF5 or Flag-HSCARG were infected with SeV for 6 h, and then IRF3 phosphorylation was analyzed with p-IRF3 antibody. (F) HSCARG suppresses IRF3 dimerization. HEK293T cells transfected with control vector or Flag-RNF5 or Flag-HSCARG were infected with SeV for 6 h, and then the effect of HSCARG on IRF3 dimerization was examined by Native PAGE. (G) HSCARG inhibits the nuclear translocation of TRAF3 triggered by viral infection. The wild-type and *HSCARG^−/^* ^−^ HeLa cells were infected by SeV (40 HAU/ml) for 0 and 6 h, and the subcellular location of endogenous IRF3 was detected with anti-IRF3 using Zeiss LSM 710&NLO. Scale bar, 10 µm.

It is known that IKKε and TBK1 are downstream kinases of TRAF3, and they are recruited to MAVS-TRAF3 complex after TRAF3 ubiquitination. Inhibition of HSCARG on TRAF3 ubiquitination drove us to further explore the effect of HSCARG on the recruitment of IKKε/TBK1. Co-IP analysis showed that endogenous HSCARG interacted with IKKε rather than TBK1 (Figure 4A, Figure S4A). Consistent with previous reports, IKKε and TRAF3 formed a complex that was stabilized by SeV infection (Figure S4C). Ectopic HSCARG impaired the interaction between TRAF3 and IKKε (Figure 4B); however, it had no effect on the stability of TRAF3-TBK1 complex (Figure S4B). On the contrary, knockdown of HSCARG increased the association of TRAF3 with IKKε (Figure 4C).

After recruitment to the TRAF3 complex, IKKε is phosphorylated and sequentially phosphorylates its substrate IRF3. As HSCARG interfered with the recruitment of IKKε, it probably dampened the phosphorylation of IKKε. To confirm this hypothesis, we investigated the effect of HSCARG on IKKε and IRF3 phosphorylation activity by performing *in vitro* phosphorylation assays. We found that HSCARG impaired both IKKε and IRF3 phosphorylation level *in vitro* (Figure 4D). It is worth mentioning that TRAF3 was found to be attached to the enriched IKKε, suggesting that TRAF3 is important for HSCARG to regulate IKKε and IRF3 phosphorylation. Consequently, similar to the RNF5 positive control (RNF5 is an ubiquitin ligase that down-regulates antiviral response through mediating the degradation of MITA [32]), HSCARG suppressed the IRF3 phosphorylation and dimerization induced by SeV *in vivo* (Figure 4E, 4F). In addition, we assessed the nuclear translocation of IRF3 in *HSCARG* ^−/−^ and wild-type HeLa cells, respectively. In response to SeV infection, more IRF3 transported into the nucleus in the *HSCARG* ^−/−^ cells, suggesting that HSCARG decreases the translocation of IRF3 (Figure 4G). Taken together, HSCARG interacts with IKKε and blocks the formation of the TRAF3-IKKε complex, which subsequently impairs the phosphorylation of IKKε and IRF3 and finally decreases IFN-β production.

### TRAF3 is essential for HSCARG to down-regulate *IFN-β*

**Figure 5:**
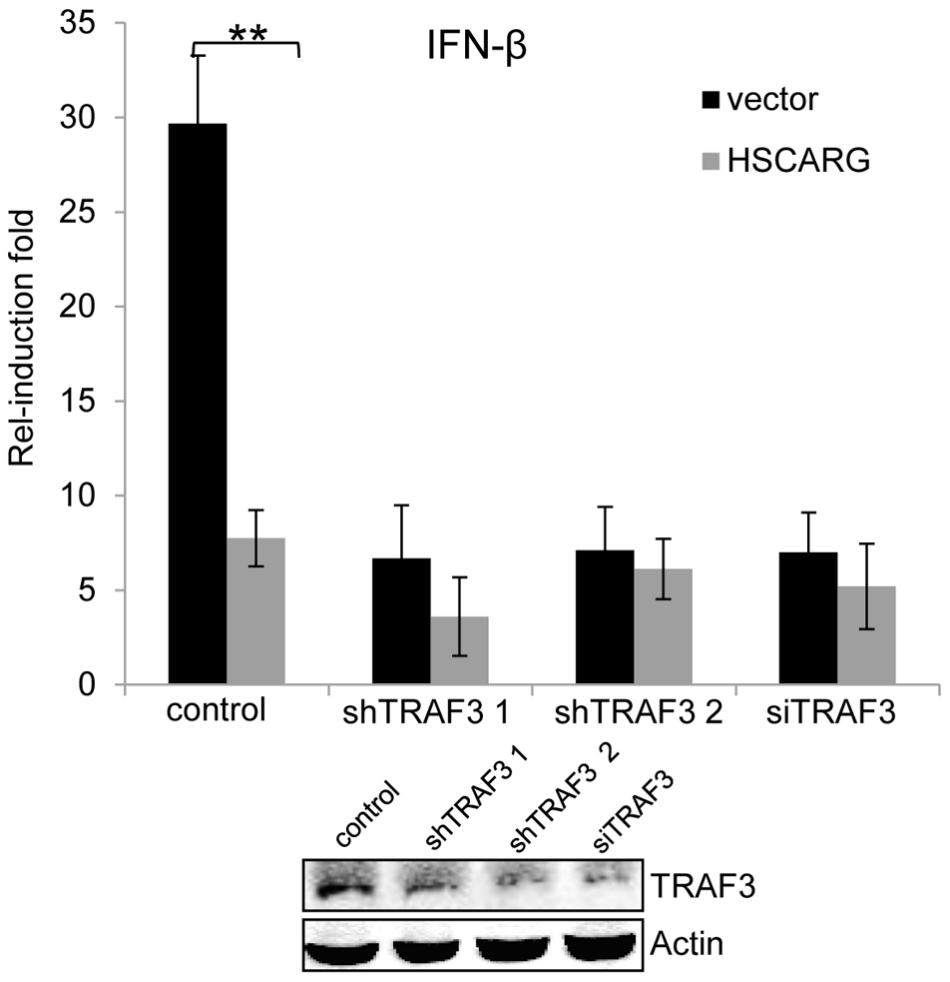
TRAF3 is necessary for HSCARG to down-regulate *IFN-β*. HEK293T wild-type cells and TRAF3 depleted cells (two stable cell lines harboring TRAF3 shRNA were obtained, or cells treated with 40 nM TRAF3 siRNA) were transfected with 200 ng *IFN-β* reporter and 20 ng pRL-TK1 plasmids, together with 600 ng vector or Flag-HSCARG, and then infected with SeV for 12 h. Luciferase reporter assay was performed to examine the activation of *IFN-β* in cells with depleted TRAF3. The knockdown effect of TRAF3 was shown at the bottom. Experiments were performed in triplicates for at least three times. The bar graphs show mean±S.D. **p<0.01.

The above data showed that HSCARG blocks TRAF3 ubiquitination and so triggers a series of downstream effects. Hence we used the stable HEK293T cells with silenced TRAF3 or TRAF3 siRNA to detect whether TRAF3 is essential for the regulatory function of HSCARG. The *IFN-β* reporter assay showed that in cells with depleted TRAF3, the induction of *IFN-β* was reduced greatly and the inhibitory effect of HSCARG was attenuated markedly (Figure 5), without affecting the stability of endogenous TRAF3 (Figure S5). These data suggested that TRAF3 is necessary for HSCARG to decrease *IFN-β* activity.

## Discussion

Previous studies have demonstrated that HSCARG is a negative regulator of TNFα-, IL-1β-induced NF-κB activity by targeting the canonical IκB kinase complex [29]. Here, we identified HSCARG as a critical component in the virus-triggered IRF3 activation pathway and the cellular antiviral response, and elucidated the underlying mechanism.

**Figure 6:**
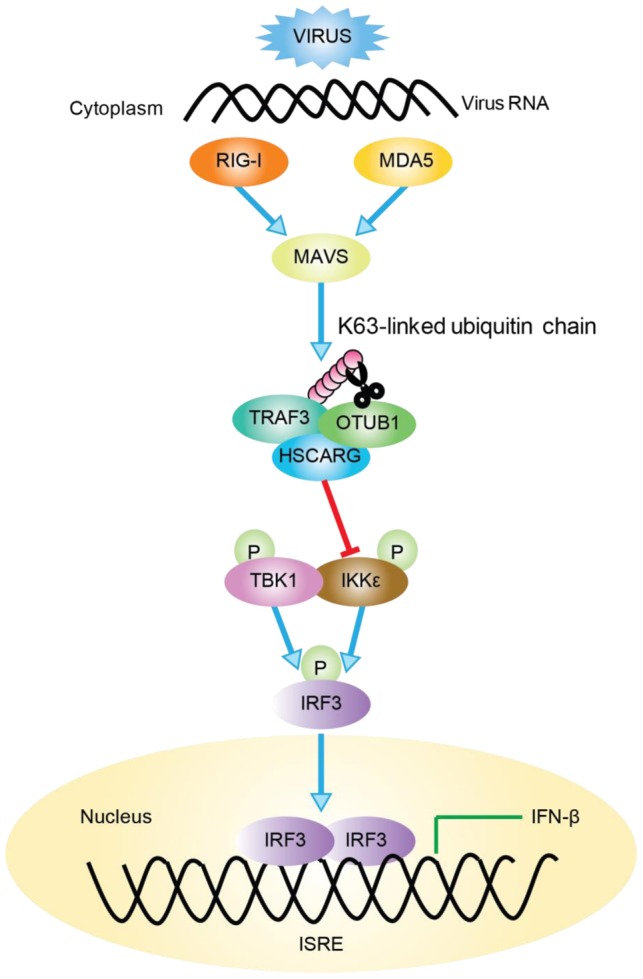
Working model of TRAF3-dependent regulation of RLR signaling pathway by HSCARG. Followed viral infection, HSCARG interacts with TRAF3, and cooperates with OTUB1 to remove Lys63-linked polyubiquitin chain from TRAF3. This further decreases the recruitment of IKKε and results in decreased phosphorylation of IKKε and IRF3, and finally reduces the production of IFN-β.

TRAF3 is a key molecule in virus-triggered IRF3 activation and IFN induction [23]; it is the pivot of the TRIF- and MyD88-dependent, TLR-independent pathways [33]. To achieve its negative regulatory function in cellular antiviral response, HSCARG associates with TRAF3 and cooperates with OTUB1 to remove TRAF3 poly-ubiquitin chain that is important for the recruitment of TBK1-IKKε kinase complex. This further decreases the recruitment of IKKε, impairs IRF3 phosphorylation and dimerization, and results in a reduced level of IFN-β transcription (Figure 6).

Recent studies have indicated that ubiquitination and de-ubiquitination play critical roles in regulating virus-triggered IFN production to assure that the antiviral response is modulated properly [34]–[36]. For example, when regulated by E3 ligases such as Triad3A and deubiquitinases including OTUB1/2, DUBA, and UCHL1, TRAF3 undergoes a biphasic ubiquitination that varies between the K63-linked and the K48-linked type in different phases after viral stimulation to maintain its activity in a suitable state [24]–[27], [37]. However, the detailed mechanism of how these regulatory proteins function together in fine-tuning the TRAF3 activity, and whether other proteins are involved in this complicated regulation remained to be discovered. Here, we found that HSCARG selectively utilized OTUB1 to suppress TRAF3 ubiquitination (Figure 3). HSCARG neither interacted with DUBA nor promoted its recruitment (Figure S6A, B), and depletion of DUBA had no effect on HSCARG in inhibiting TRAF3 ubiquitination (Figure S6C). These data suggested that HSCARG specifically relies on OTUB1 to repress TRAF3 ubiquitination. Based on this, it is reasonable to speculate that the stimulus-specific regulation of TRAF3 ubiquitination is achieved through recruiting distinct E3 ligases or deubiquitinases mediated by specific adaptor proteins. Our data suggest that HSCARG is such an adaptor protein, which cooperates with a specific deubiquitinase to remove the TRAF3 ubiquitin chain in response to a viral stimulus, and to achieve accurate and timely modulation of TRAF3 activity.

In response to different PAMPs, NF-κB and IRF3 promote pro-inflammatory cytokines and type I IFN production to initiate innate immunity [1]. There is close crosstalk between these two pathways at different steps to ensure a balanced and robust defense. For example, NEMO is not only the modulating subunit for the canonical IKK complex but also participates in the TBK1-IKKε complex [38]. Our previous studies demonstrated that HSCARG plays a critical role in TNFα- and IL-1β-induced NF-κB signaling. In this study, we further characterized the function of HSCARG in IRF3 signaling in response to viral invasion. Taken together, we propose that HSCARG functions *via* cross-talk to coordinate the activities of NF-κB and IRF3, and to balance the production of pro-inflammatory cytokines and type I interferons. In response to different stimuli, HSCARG regulates the innate immune response either by inhibiting the NF-κB activity mediated by the canonical IKK complex or by modulating the IRF3 activity mediated by TRAF3-IKKε.

Virus-triggered IFNs induction is important for the innate antiviral response. The intensity and duration of the antiviral response must be limited to an optimal range; otherwise it could cause inflammatory damage and finally lead to autoimmune disease. The production of IFN-β and relevant pro-inflammatory cytokines is regulated delicately through various molecules and distinct mechanisms in a specific spatiotemporal manner. Here we demonstrated that HSCARG plays an important role in the precise control of the cellular antiviral response by negatively regulating TRAF3 ubiquitination and IFN-β production, providing a potential target for the treatment of chronic inflammation and autoimmune disease.

## Materials and Methods

### Reagents and antibodies

Cells were transfected with the indicated plasmids using PEI (Polyscience, USA) following the manufacturer's instructions. Screen FectA (S-3001) (InCella, Germany) was used for shRNA or siRNA transfection. The following commercial antibodies were used: monoclonal anti-Flag (F3165) and anti-HA (H9658) from Sigma (USA); anti-Myc (M047-3), anti-His (D291-3) and anti-β-actin (PM053) from MBL (Japan); anti-P-IRF3 pS386 (2562-1) and anti-TRAF3 (3555-1) from EPITOMICS (USA); anti-p65 (4764) from Cell Signaling Tech (USA). Mouse polyclonal antibody against HSCARG and IRF3 were generated by immunizing mice with purified HSCARG and IRF3 proteins.

### Plasmids, cell-lines, and viruses

*NF-κB*, *IFN-β*, and *ISRE* promoter reporter plasmids, mammalian expression plasmids for Flag-RIG-I-N, Flag-MDA5-N, HA-MAVS, HA-TRIF, Flag-TBK1 and Flag-IKKε were kind gifts from Profs. Zhengfan Jiang and Danying Chen; Flag-OTUB1 and Flag-OTUB2 were kind gifts from Prof. Hongbing Shu; Flag-USP25 was from Drs. Dong Chen and Bo Zhong; Flag-OTUD7B was from Dr. Hsiu-Ming Shih. HSCARG expression vectors tagged with Flag, HA or Myc were constructed previously. His-ubiquitin and its mutants *K63R*, *K48R*, *K63*, and *K48* were cloned to pEF1-HisA. HA-TRAF3 was sub-cloned to pCMV-HA, Flag-TRAF3 truncation constructs 1–108, 109–347, and 348–568 were inserted into pRK-Flag.

The *HSCARG* ^−/−^ HCT116 cell-line was generated using the Cre/loxP system by inserting an additional sequence into the fourth exon and additional stop codons to disrupt HSCARG translation. The *HSCARG* ^−/−^ HEK 293T and HeLa cell-line was generated by the TALEN method using the FastTALE™ TALEN Assembly Kit (SIDANSAI). TRAF3 knockdown HEK293T cells were generated by transfecting TRAF3 shRNA and selecting with puromycin (2 µg/ml) for 3 weeks. HEK293T and HCT116 cells were cultured in Iscove's modified Dulbecco's medium (Hyclone, USA) with 10% fetal calf serum (Genstar, China) at 37°C in a 5% CO_2_ incubator.

Sendai virus was from Prof. Danying Chen, propagated in chicken embryos, and titrated by the hemagglutination test. Cells were infected at 40 HAU/ml for the indicated times. Vesicular stomatitis virus (VSV) was from Prof. Zhengfan Jiang and was titrated by the plaque assay. Cells were infected at an MOI of 1.

### *IFN-β* Dual-luciferase reporter assay

HEK293T cells (1×10^5^) were seeded on a 24-well plate and transfected with the *IFN-β firefly* luciferase reporter plasmid, *Renilla* reporter plasmid, and other plasmids as indicated. 24 h later, cells were harvested and luciferase assay was performed using the Dual-luciferase reporter assay system (Promega). All the experiments were performed in triplicate and repeated at least three times. Data shown are mean ± S.D. from one representative experiment.

### RNAi

The siRNA cocktail for p65 (sc-29140), OTUB1 (sc-76014) and TRAF3 (sc-29510) were purchased from Santa Cruz (USA). TRAF3 shRNA plasmids were chosen from Sigma TRC1.0 shRNA library. The targeted sequences of shRNA used are as follow. TRAF3 shRNA 2: 5′-tgtcaagagagcatcgtta; TRAF3 shRNA 4: 5′-ttggccgtttaagcagaaa; HSCARG shRNA: 5′- accttcatcgtgaccaatt; DUBA shRNA: 5′-cagtggtgaatcctaacaa. The non-silencing sequences were used as negative controls.

### RT-PCR

HEK293T (1×10^5^) cells were seeded on a 6-well plate and infected with SeV at 40 HAU/ml for indicated time. Cells were harvested and the mRNAs were extracted with Trizol (Invitrogen). The primers used are as follow.

*HSCARG*: 5′-gaagctgctcgctgatctg (forward), 5′-aaggctgagcaccacagga (reverse); *IFN-β*: 5′-ccaacaagtgtctcctccaa (forward), 5′-atagtctcattccagccagt (reverse); *Actin*: 5′-aagtgtgacgtggacatccgc (forward), 5′-ccggactcgtcatactcctgct (reverse).

### Vesicular stomatitis virus (VSV) plaque assay

HEK293T cells seeded in 24-well plates were transiently transfected with indicated plasmids. 24 h later, cells were washed gently with preheated PBS and infected by VSV at an MOI of 1 for 1 h. Cells were then washed and cultured in fresh medium for 12 or 24 h. The supernatants were collected and diluted from 1∶10 to 1∶10^8^, and infected the confluent BHK21 cells at a dilution from 1∶10^6^ to 1∶10^8^ at 37°C for 1 h. After washing with preheated PBS again, wells were covered with 1.5% methylcellulose DMEM. 3 days later, cells were fixed in 0.5% glutaraldehyde for 30 min and stained with 1% crystal violet dissolved in 70% ethanol. Plaques were counted, averaged, and multiplied by the dilution factor to determine viral titer as Pfu/ml. The experiments were performed in triplicate for three times. Data shown are mean ± S.D. from one representative experiment.

### Native PAGE

HEK293 cells (2×10^5^) were seeded on a 12-well plate and transfected with indicated plasmids. 24 h later, cells were harvested with cold PBS and lysed by 30 µl 1.5% TritonX-100 lysis buffer for 30 min on ice, and then centrifuged at 14000 rpm for 20 min. The supernatants were mixed with 30 µl loading buffer (62.5 mM Tris-HCl, pH 6.8, 15% glycerol and 1% deoxycholate). Samples were applied to a 8% native acrylamide gel (free of SDS) that was pre-run at 40 mA for 30 min in running buffer (25 mM Tris and 192 mM glycine, pH 8.4) with or without 1% deoxycholate for the cathode and anode chamber, respectively. And then the samples were electrophoresed for 60 min at 25 mA in 4°C.

### ELISA

HEK293T cells were seeded on a 24-well plate and transfected with the indicated plasmids for triplicates the following day. At 18 h after transfection, cells were infected with SeV (40 HAU/ml) for 12 h, and then collected the supernatant and ELISA was performed using human interferon β ELISA kit (RD, USA).

### Co-immunoprecipitation (Co-IP) and quantification of gel image

HEK293T cells were transfected with the indicated plasmids and harvested at 48 h after transfection. Then Co-IP analyses were performed following the procedure described previously [39]. The band intensity of interest was quantified using Odyssey Infrared Imaging System and software Odyssey V3.0 (LI-COR Biosciences, USA) and normalized to corresponding enriched protein amount.

### His-ubiquitin pull-down assay

HEK293T cells were transfected with His-ubiquitin and relevant plasmids for 24 h. Cells were harvested and His-ubiquitin pull-down analysis was performed following the procedures described previously [39].

### IKKε *in vitro* kinase assay

GST-tagged IRF3 (131–426) and His-tagged HSCARG were purified from *E. coli BL21(DE3)*, Flag-IKKε was immune-precipitated with anti-Flag antibody. Reaction mixture contained 2 µg GST-IRF3, enriched IKKε, 0.5 µCi [γ-^32^P]-ATP, and 20 µl kinase reaction buffer (1 mM DTT, 50 mM KCl, 2 mM MgCl_2_, 2 mM MnCl_2_, 10 mM NaF, 1 mM Na_3_VO_4_, 25 µM ATP). The reaction solution was incubated at 25°C for 30 min, and stopped by adding 20 µl loading buffer, and then separated by SDS-PAGE and auto-radiographed using a storage phosphor screen (GE).

### Data statistical analysis

Each experiment was performed in triplicates and repeated at least three times, and values were represented as mean ± S.D. The student's *t*-test was used to determine the difference between experimental and control groups, requiring *p*<0.05 for statistical significance.

### Proteins accession numbers

The accession numbers in the UniProtKB/SwissProt database for the proteins in the manuscript are as followed. HSCARG, Q9HBL8; TRAF3, Q13114; RIG-I, O95786; MDA5, Q9BYX4; MAVS, Q7Z434; PCBP2, Q15366; TBK1, Q9UHD2; IKKε, Q14164; IRF3, Q14653; IRF7, Q92985; RNF5, Q99942; OTUB1, Q96FW1; OTUB2, Q96DC9; UCHL1, P09936; DUBA, Q96G74; USP25, Q9UHP3.

## Supporting Information

Figure S1 **HSCARG inhibits** ***IFN-β*** **activity specifically and does not affect the stability of RLR adaptors.** (A) The inhibition of *IFN-β* by HSCARG is specific. HEK293T cells (1×10^5^) transfected with increasing dose of HSCARG and the negative control STK38 (200, 400 and 800 ng) were infected with SeV (40 HAU/ml) for 12 h, and then luciferase reporter assays was performed to examine the activity of *IFN-β*. (B) HSCARG does not affect the stability of RLR adaptors. The samples of the luciferase reporter assay of Figure 1B were subjected to western blot to detect the effect of HSCARG on the expression level of RLR adaptors with corresponding antibodies.
(TIF)

Figure S2 **Inhibition of NF-κB has no distinct effect on HSCARG in regulating** ***IFN-β*** **activity.** (A) HEK293T cells (1×10^5^) were transfected with p65 siRNA (30 nM) or control siRNA prior to SeV infection, and then luciferase reporter assay was performed to detect the activation of *IFN-β* and *ISRE*. The knockdown effect of p65 was confirmed by western blot. (B) HEK293T cells transfected with indicated plasmids were treated with 1 mM of DMSO or PDTC (a specific inhibitor of NF-κB) for 2 h, and then infected with SeV for 18 h. Luciferase reporter assay was then performed to detect *IFN-β* and *ISRE* activity. The PDTC inhibition effect was shown in the right. All experiments were performed in triplicate for at least three times with similar results. The data represent the mean±S.D. **p<0.01.
(TIF)

Figure S3 **TRAF3 is the potential target of HSCARG, and HSCARG interacts most potently with OTUB1.** (A) HSCARG interacts with TRAF3 most potently. HEK293T cells were transfected with Flag-RIG-I, HA-MDA5, HA-MAVS, Flag-TRAF3, Flag-TBK1, Flag-IKKε, Flag-IRF3, Flag-IRF7 in order and plus Myc-HSCARG, and then IP was performed with anti-Flag followed by IB with anti-Myc and anti-Flag antibodies. (B) HSCARG interacts strongly with OTUB1. HEK293T cells transfected with indicated plasmids were subjected to Co-IP analysis to examine the interaction between HSCARG and Flag-OTUB1, Flag-OTUD7B, Flag-USP25. (C) Knockout of HSCARG impairs the negative regulation of *IFN-β* by OTUB1. The wild-type or *HSCARG* ^−/−^ HEK293T cells (1×10^5^) were transfected with OTUB1 siRNA (40 nM) prior to SeV infection, and then luciferase reporter assay was performed to detect the activation of *IFN-β*. The knockdown effect of OTUB1 was confirmed by western blot analysis. This experiment was repeated three times and the data represent mean±S.D. **p<0.01.
(TIF)

Figure S4 **HSCARG does not interact with TBK1 but can attenuate the IKKε-TRAF3 interaction stimulated by SeV infection.** (A) HSCARG does not interact with TBK1. HEK293T cells transfected with Flag-TBK1 and Myc-HSCARG were IP with anti-Flag followed by IB with anti-Myc and anti-Flag antibodies. (B) HSCARG does not impair TRAF3-TBK1 interaction. HEK293T cells transfected with indicated plasmids were subjected to Co-IP analysis to examine the effect of HSCARG on the TBK1-TRAF3 interaction. (C) HSCARG inhibits the virus-triggered TRAF3-IKKε interaction. HEK293T were transfected with HA-TRAF3 and Flag-IKKε with or without Myc-HSCARG, and stimulated with SeV for 0, 2, 12, 24, and 48 h, and then Co-IP analysis was performed to detect the effect of HSCARG on the interaction between TRAF3 and IKKε.
(TIF)

Figure S5 **HSCARG does not affect the stability of TRAF3.** HEK293T cells transfected with vector or Flag-HSCARG were infected with SeV for 0, 12, 24, 36, 48, and 72 h, and then the endogenous level of TRAF3 was detected by anti-TRAF3 antibody.
(TIF)

Figure S6 **DUBA is not essential for HSCARG in inhibition of TRAF3 ubiquitination.** (A) HSCARG does not associate with DUBA. HEK293T cells transfected with Flag-DUBA and HA-HSCARG were subjected to Co-IP analysis. (B) HSCARG does not promote the recruitment of DUBA. HEK293T cells were transfected with Myc-HSCARG or HSCARG shRNA and other indicated plasmids for 72 h, and then subjected to Co-IP to detect the effect of HSCARG on TRAF3-DUBA interaction. (C) HSCARG does not rely on DUBA to inhibit TRAF3 ubiquitination. HEK293T cells were transfected with negative siRNA control or DUBA siRNA (50 nM) and other indicated plasmids for 72 h, and then His-ubiquitin pull-down analysis was performed to monitor TRAF3 ubiquitination level.
(TIF)
