# Supplementary figures and images for: HSCARG Negatively Regulates the Cellular Antiviral RIG-I Like Receptor Signaling Pathway by Inhibiting TRAF3 Ubiquitination via Recruiting OTUB1
Source: PLoS Pathog. 2014 Apr 24;10(4):e1004041. doi: 10.1371/journal.ppat.1004041 (PMC3999155; doi:10.1371/journal.ppat.1004041)

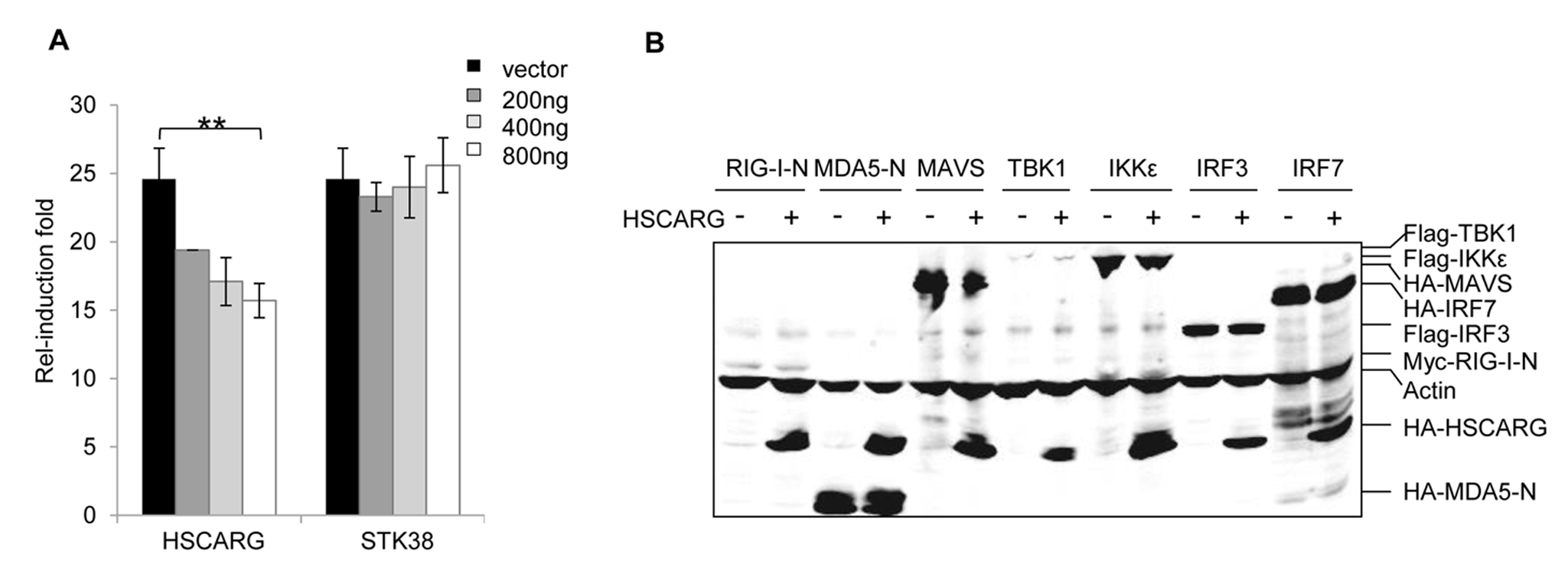

Supplement: Figure S1 — HSCARG inhibits IFN-β activity specifically and does not affect the stability of RLR adaptors. (A) The inhibition of IFN-β by HSCARG is specific. HEK293T cells (1×10^5^) transfected with increasing dose of HSCARG and the negative control STK38 (200, 400 and 800 ng) were infected with SeV (40 HAU/ml) for 12 h, and then luciferase reporter assays was performed to examine the activity of IFN-β. (B) HSCARG does not affect the stability of RLR adaptors. The samples of the luciferase reporter assay of Figure 1B were subjected to western blot to detect the effect of HSCARG on the expression level of RLR adaptors with corresponding antibodies. (TIF) [file ppat.1004041.s001.tif]

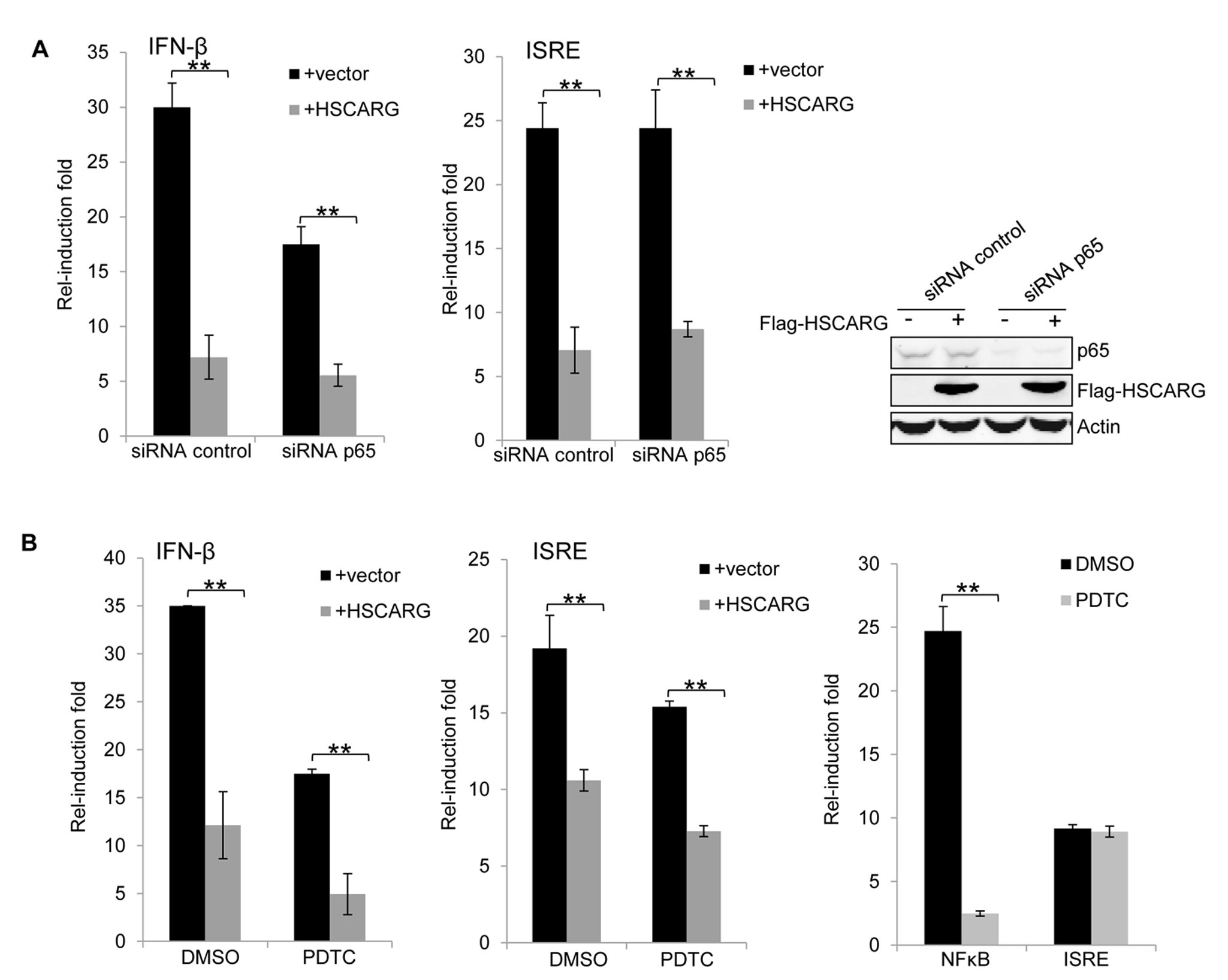

Supplement: Figure S2 — Inhibition of NF-κB has no distinct effect on HSCARG in regulating IFN-β activity. (A) HEK293T cells (1×10^5^) were transfected with p65 siRNA (30 nM) or control siRNA prior to SeV infection, and then luciferase reporter assay was performed to detect the activation of IFN-β and ISRE. The knockdown effect of p65 was confirmed by western blot. (B) HEK293T cells transfected with indicated plasmids were treated with 1 mM of DMSO or PDTC (a specific inhibitor of NF-κB) for 2 h, and then infected with SeV for 18 h. Luciferase reporter assay was then performed to detect IFN-β and ISRE activity. The PDTC inhibition effect was shown in the right. All experiments were performed in triplicate for at least three times with similar results. The data represent the mean±S.D. **p<0.01. (TIF) [file ppat.1004041.s002.tif]

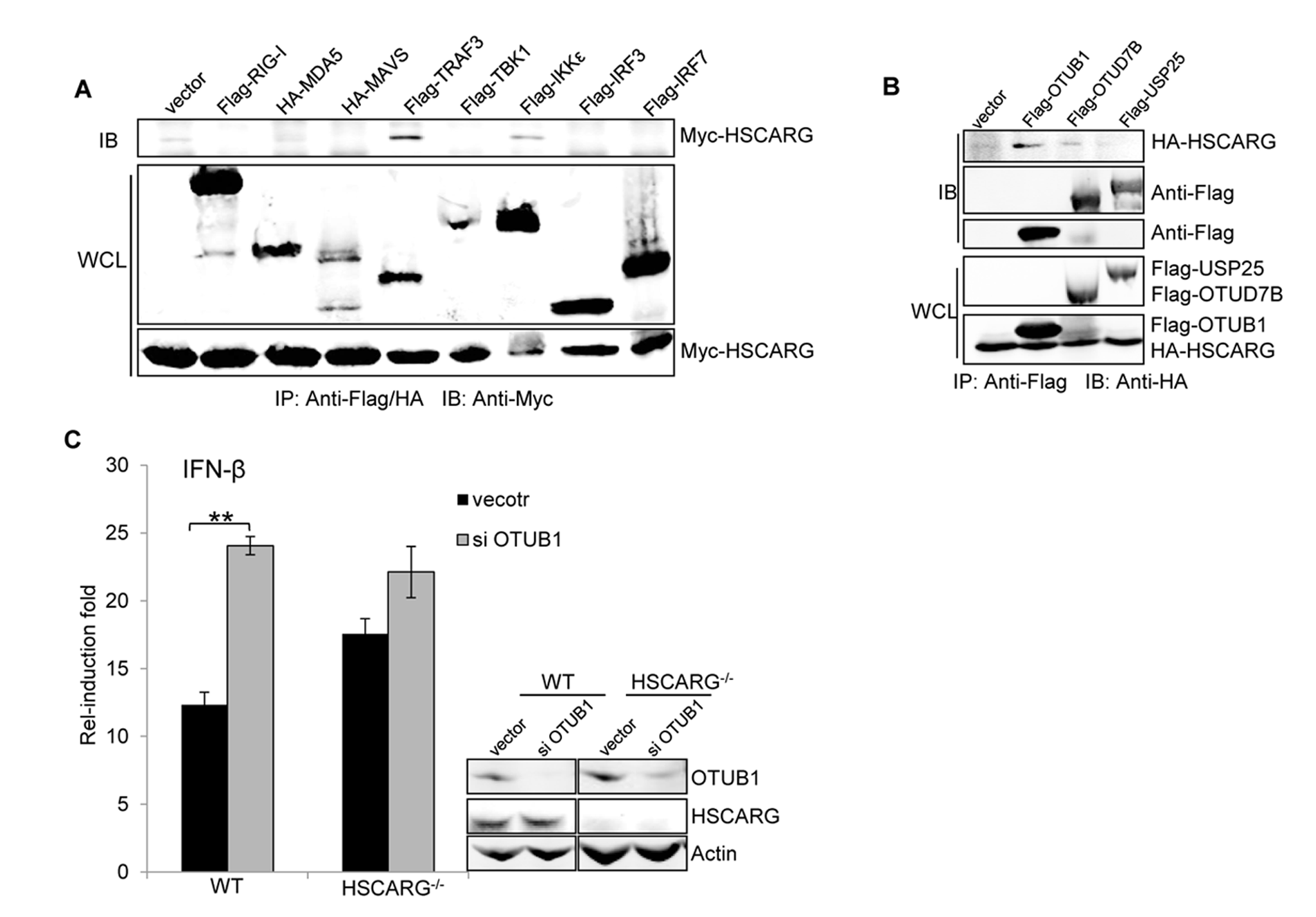

Supplement: Figure S3 — TRAF3 is the potential target of HSCARG, and HSCARG interacts most potently with OTUB1. (A) HSCARG interacts with TRAF3 most potently. HEK293T cells were transfected with Flag-RIG-I, HA-MDA5, HA-MAVS, Flag-TRAF3, Flag-TBK1, Flag-IKKε, Flag-IRF3, Flag-IRF7 in order and plus Myc-HSCARG, and then IP was performed with anti-Flag followed by IB with anti-Myc and anti-Flag antibodies. (B) HSCARG interacts strongly with OTUB1. HEK293T cells transfected with indicated plasmids were subjected to Co-IP analysis to examine the interaction between HSCARG and Flag-OTUB1, Flag-OTUD7B, Flag-USP25. (C) Knockout of HSCARG impairs the negative regulation of IFN-β by OTUB1. The wild-type or HSCARG −/− HEK293T cells (1×10^5^) were transfected with OTUB1 siRNA (40 nM) prior to SeV infection, and then luciferase reporter assay was performed to detect the activation of IFN-β. The knockdown effect of OTUB1 was confirmed by western blot analysis. This experiment was repeated three times and the data represent mean±S.D. **p<0.01. (TIF) [file ppat.1004041.s003.tif]

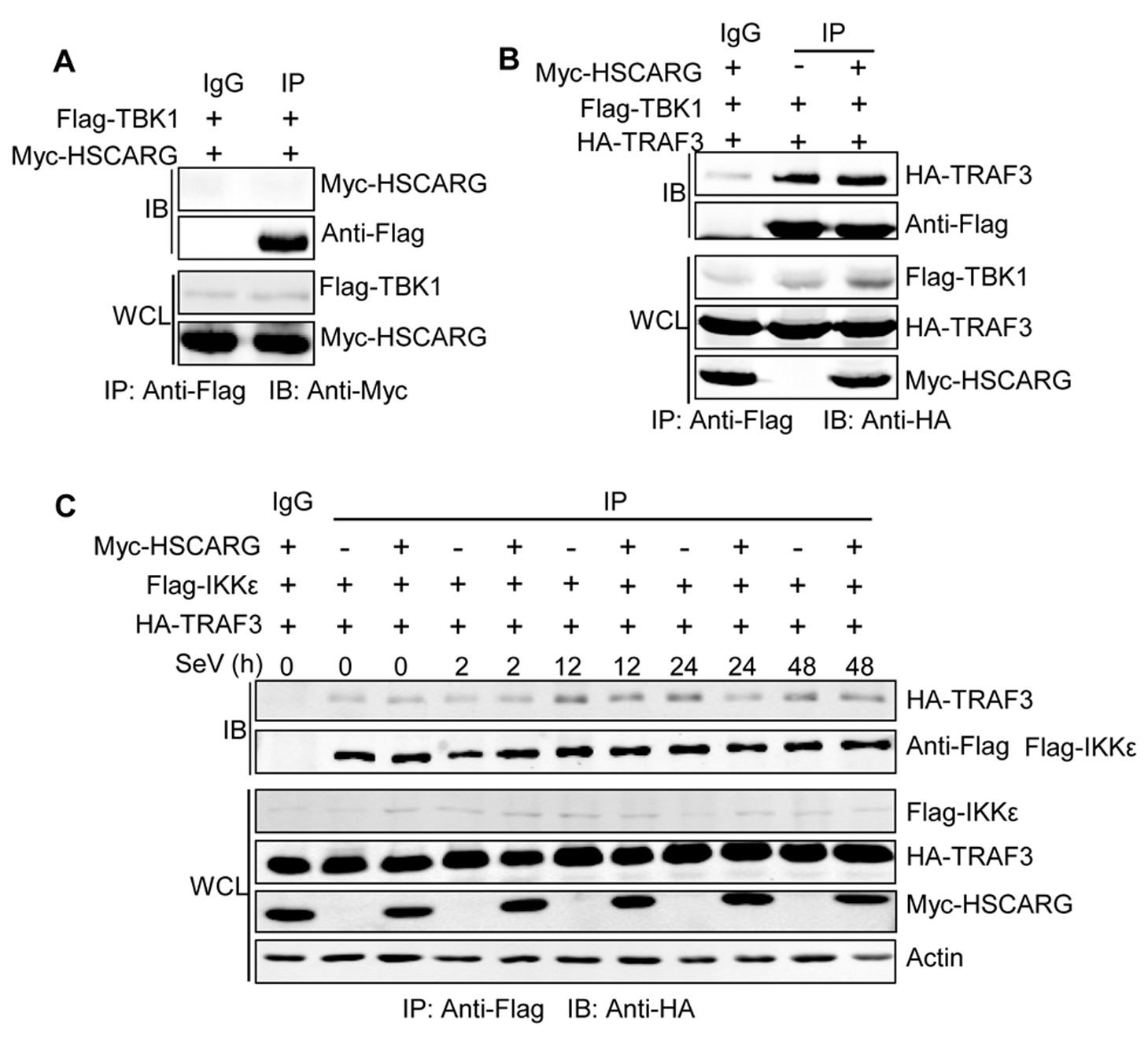

Supplement: Figure S4 — HSCARG does not interact with TBK1 but can attenuate the IKKε-TRAF3 interaction stimulated by SeV infection. (A) HSCARG does not interact with TBK1. HEK293T cells transfected with Flag-TBK1 and Myc-HSCARG were IP with anti-Flag followed by IB with anti-Myc and anti-Flag antibodies. (B) HSCARG does not impair TRAF3-TBK1 interaction. HEK293T cells transfected with indicated plasmids were subjected to Co-IP analysis to examine the effect of HSCARG on the TBK1-TRAF3 interaction. (C) HSCARG inhibits the virus-triggered TRAF3-IKKε interaction. HEK293T were transfected with HA-TRAF3 and Flag-IKKε with or without Myc-HSCARG, and stimulated with SeV for 0, 2, 12, 24, and 48 h, and then Co-IP analysis was performed to detect the effect of HSCARG on the interaction between TRAF3 and IKKε. (TIF) [file ppat.1004041.s004.tif]

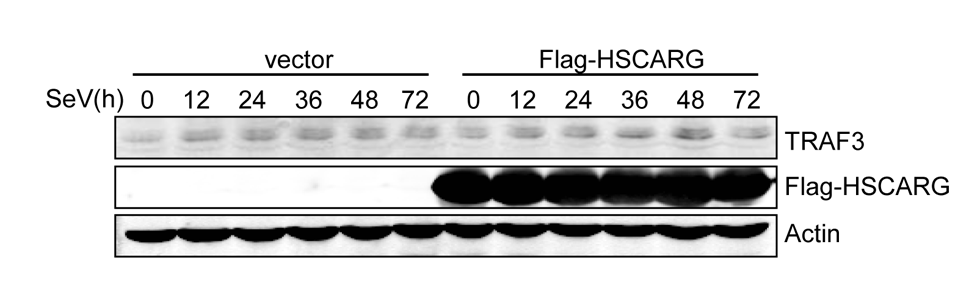

Supplement: Figure S5 — HSCARG does not affect the stability of TRAF3. HEK293T cells transfected with vector or Flag-HSCARG were infected with SeV for 0, 12, 24, 36, 48, and 72 h, and then the endogenous level of TRAF3 was detected by anti-TRAF3 antibody. (TIF) [file ppat.1004041.s005.tif]

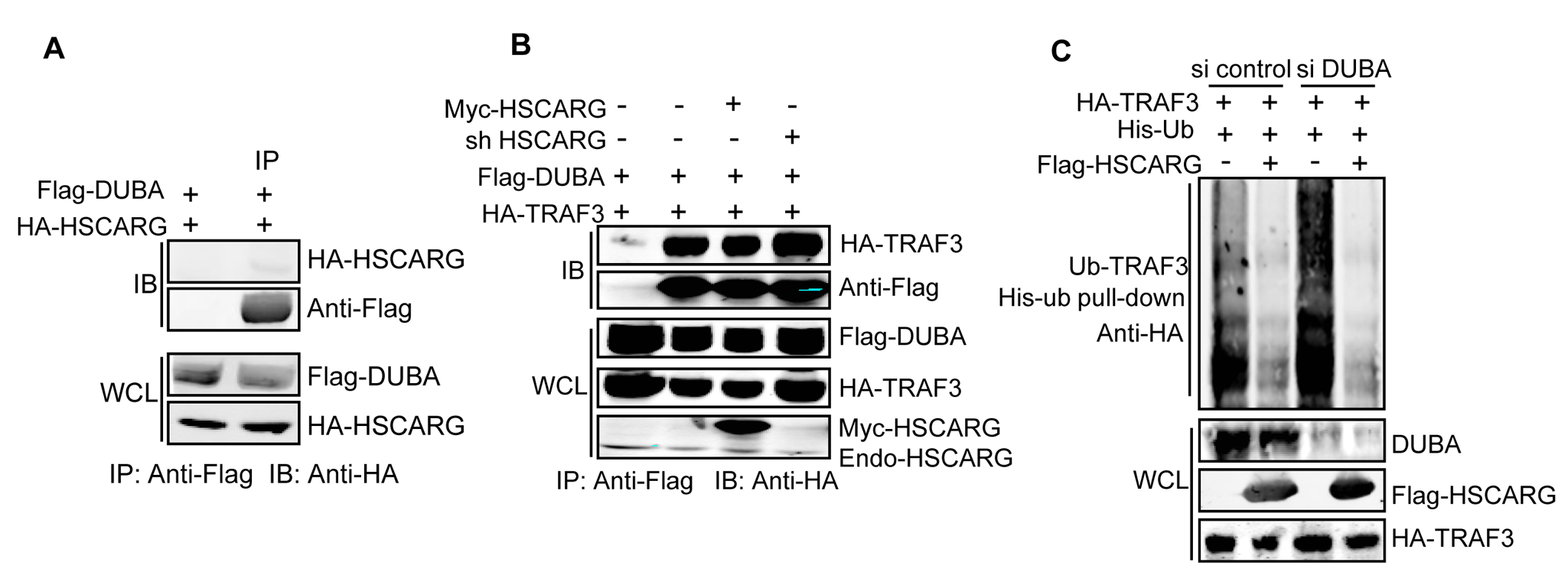

Supplement: Figure S6 — DUBA is not essential for HSCARG in inhibition of TRAF3 ubiquitination. (A) HSCARG does not associate with DUBA. HEK293T cells transfected with Flag-DUBA and HA-HSCARG were subjected to Co-IP analysis. (B) HSCARG does not promote the recruitment of DUBA. HEK293T cells were transfected with Myc-HSCARG or HSCARG shRNA and other indicated plasmids for 72 h, and then subjected to Co-IP to detect the effect of HSCARG on TRAF3-DUBA interaction. (C) HSCARG does not rely on DUBA to inhibit TRAF3 ubiquitination. HEK293T cells were transfected with negative siRNA control or DUBA siRNA (50 nM) and other indicated plasmids for 72 h, and then His-ubiquitin pull-down analysis was performed to monitor TRAF3 ubiquitination level. (TIF) [file ppat.1004041.s006.tif]
